# Green and recyclable catalyst based on chitosan/CuFe_2_O_4_ nanocomposite hydrogel for one-step synthesis of 1,2,3-triazoles†

**DOI:** 10.1039/d4ra05626d

**Published:** 2024-10-01

**Authors:** Fatemeh Ajormal, Rahman Bikas, Hossein Ghasemzadeh, Nader Noshiranzadeh, Anna Kozakiewicz-Piekarz

**Affiliations:** a Department of Chemistry, Faculty of Science, University of Zanjan Zanjan 45371-38791 Iran; b Department of Chemistry, Faculty of Science, Imam Khomeini International University Qazvin 34148-96818 Iran hoghasemzadeh@sci.ikiu.ac.ir bikas@sci.ikiu.ac.ir; c Department of Biomedical and Polymer Chemistry, Faculty of Chemistry, Nicolaus Copernicus University in Torun Torun 87-100 Poland

## Abstract

The scope of the heterogeneous catalysts has been greatly expanded in last few decades by the development of various catalysts. In this work a new chitosan-based nanocomposite hydrogel (CS/CuFe_2_O_4_ NCH) was synthesized as a high-performance heterogeneous catalyst and then, it was utilized for the green synthesis of substituted 1,2,3-triazoles by a multi-component (azide–alkyne-epoxide) cycloaddition reaction. The synthesized nanocomposite hydrogel was investigated by using various instrumental analyses, including FT-IR, XRD, SEM, EDS, HRTEM, DLS, and TGA. The structure of one of the substituted 1,2,3-triazoles was studied by using single-crystal X-ray diffraction analysis. The nanocomposite hydrogel can be easily regenerate after the catalytic reaction. It can be reused frequently without considerable loss of activity. The high catalytic activity, straightforward reaction, easy recyclability, short reaction time, use of a green solvent, and the simple separation of catalyst are the main advantage of the current method, which offers both financial and environmental benefits.

## Abstract 2

Synthesis and characterization of a new recyclable CS/CuFe_2_O_4_ nanocomposite hydrogel and its catalytic activity in the green synthesis of 1,2,3-triazoles have been reported.

## Graphical abstract

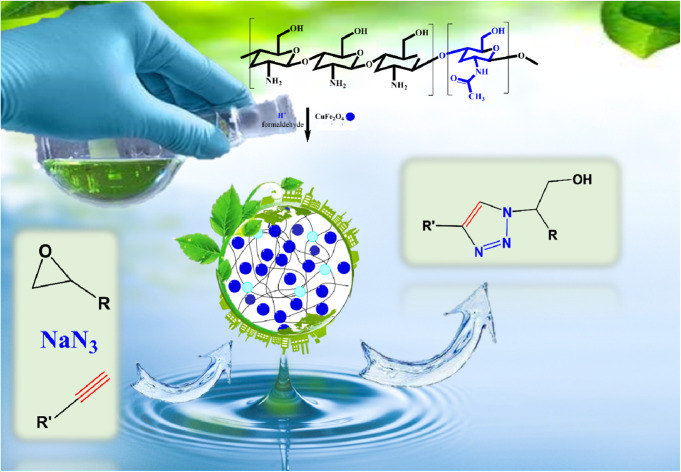

## 1. Introduction

Green chemistry has received much attention in recent years, mostly for environmental and economic reasons. The replacement of petroleum-based materials with natural-source materials has become a significant need.^1,2^ This demand increases when it comes to items that are related to human health, such as medical, pharmaceutical, and food products.^3^ One important aspect of the green chemistry, that has received high attention, is the manufacture of the active green catalytic systems. Designing a safe, low-cost, and stable heterogeneous catalytic system based on natural products represents an effective strategy for preparing environmentally friendly catalytic systems.^4,5^ Hydrogels are polymeric networks that can serve as water absorbents due to their hydrophilic nature.^6,7^ Hydrogels can be synthesized using natural materials. Most of the natural based hydrogels can be considered as green materials. Hydrogels offer a broad range of applications due to their physicochemical properties, such as excellent solvent compatibility, and porous structure.^8–11^ Hydrogels are also interesting and useful materials for preparing nanocomposites. A range of nanocomposites hydrogels have been prepared successfully.^12^ They have been utilized in several fields, including biomedicine,^13–15^ antibacterial studies,^16,17^ water treatment,^18,19^ drug delivery,^20–22^*etc.* The synthesis, applications, and various properties of the nanocomposite hydrogels have been reported by several review articles.^23–25^ Nano-composite hydrogels have also been used as catalysts in various reactions, including C–C coupling,^26,27^ reduction of organic materials,^28–31^ and oxidation of alcohols.^32^

1,2,3-Triazoles are a class of nitrogen-rich heterocyclic compounds that are commonly used in several applications such as sensors,^33^ herbicides,^34^ fungicides,^35–37^ corrosion-resistant agents,^38^ solar cells and dyes,^39^ and catalysis.^40^ Due to various biological activities, including anti HIV,^41,42^ anti bacterial,^43^ and anti allergy^44^ properties, they are also important compounds in the preparation of novel drugs and development their activities. Therefore, green, and effective synthesis of triazoles has attracted considerable attention in recent years.^45,46^ Several techniques have been used for the synthesis of triazoles. The thermal cycloaddition reaction of azides with alkynes in the presence of copper catalysts is one the most interesting methods.^47–50^ Although the primary approaches were based on the use of active Cu(i) species,^51,52^ several works have reported the direct use of Cu(ii) species as a catalyst for synthesis of triazoles.^53,54^ In addition, β-hydroxy-1,2,3-triazoles has been synthesized by *in situ* ring opening of epoxide in the presence of sodium azide and an alkyne.^55^ Several catalytic systems have also been developed for the synthesis of 1,4-disubstituted-1,2,3-triazoles.^56–59^ Several studies have been carried out to prepare green and reusable systems for the synthesis of triazoles.^60–62^ Although a few works were reported about the synthesis of triazoles by use of nanocomposite hydrogels,^63,64^ new catalytic systems based on the nanocomposite hydrogels can be developed for green synthesis of triazoles under mild conditions.

In this work, a nanocomposite hydrogel was synthesized based on the chitosan, formaldehyde, Cu(NO_3_)_2_·3H_2_O, and Fe(NO_3_)_3_·9H_2_O and used successfully as an effective catalyst for the green synthesis of 1,2,3-triazoles by an azide-epoxide-alkyne cycloaddition reaction. Chitosan is a valuable starting compound for the synthesis of hydrogels due to its unique properties, including non-toxicity, hydrophilic nature, renewability, and chelating properties.^65^ The obtained nanocomposite hydrogel was characterized by several instrumental methods. The results demonstrated that the catalytic system has great stability and recycling efficiency for the production of triazole in aqueous solution. The synthesized catalytic system can be considered as a low-cost green catalyst for the synthesis of triazoles.

## 2. Experimental

### 2.1 Materials

Formaldehyde, sodium hydroxide (NaOH), ferric nitrate (Fe(NO_3_)_3_·9H_2_O), copper(ii) nitrate (Cu(NO_3_)_2_·3H_2_O), sodium azide, propylene oxide, phenylacetylene, 2-methylbut-3-butyn-2-ol, 1-ethynylcyclohexanol, 2-(phenoxymethyl)oxirane and solvents were purchased from Sigma-Aldrich Co. High molecular weight chitosan (CS), with deacetylation degree (DD) of 85% was prepared from Sigma-Aldrich Co. All chemicals were used without further purification. Distilled water (DW) was also used in all experiments.

### 2.2 Preparation of hydrogel and nanocomposite hydrogel CS/CuFe_2_O_4_

The hydrogel was prepared according to the reported procedure.^66^ To create the chitosan hydrogel, 0.4 g of chitosan was poured to 40 mL distilled water, followed by addition of 2.0 mL acetic acid. The mixture was stirred for 10 min at 80 °C to achieve a homogenous solution. Then, formaldehyde (2.0 mL) was added to the solution. The solution was stirred for 10 min at 80 °C. After the formation of low-strength hydrogels, the product was permitted to reach to room temperature. CuFe_2_O_4_ nanoparticles were prepared within the hydrogel by *in situ* co-precipitation of copper(ii) nitrate and iron(iii) nitrate in aqueous solution. First, Cu(NO_3_)_2_·3H_2_O (2.0 mmol, 0.49 g), and Fe(NO_3_)_3_·9H_2_O (4.0 mmol, 0.82 g) were dissolved in 5.0 mL deionized water in two different flasks. After full dissolving, the solutions were combined and poured in a 20 mL flask and the hydrogel was placed in the solution. The hydrogel remained in the solution at room temperature for 24 h. Then, the hydrogel was treated dropwise with NaOH solution (20.0 mL, 0.04 M) for 30 min. Then, the mixture was heated to 45–50 °C for 30 min. After cooling, the gel containing nanoparticles was filtered and rinsed with 200 mL of deionized water. The nanocomposite hydrogel (CS/CuFe_2_O_4_) was then placed in 100 mL of ethanol and stirred at room temperature overnight to remove the water. The nanocomposite hydrogel was then filtered, and the sample was dried at 40 °C in a vacuum condition for 24 h.

### 2.3 Hydrogel characterization

Fourier transform infrared (FT-IR) spectra were obtained using KBr pellets on a Bruker Tensor 27 spectrophotometer. HR-TEM (FEI TECNAI F20TEM) and dynamic light scattering (DLS) by VASCO Cordouan technologies were used to determine the size of the nanoparticles. The CS/CuFe_2_O_4_ nanocomposite hydrogel was dispersed in distilled water by an ultrasonic homogenizer for 20 min. The morphology of the nanocomposite hydrogel was investigated by FE-SEM (TESCAN MIRA3) after freeze-drying. X-ray diffraction (XRD) pattern was studied using an XRD Philips PW1730 diffractometer with Cu-Kα radiation in the scan range of 2*θ* = 10–80° at ambient temperature.

### 2.4 Water absorbency measurement

The water absorbency of the hydrogel was examined in distilled water at ambient temperature and gentle stirring (150 rpm). In brief, 3 samples with a certain amount of dry hydrogel (0.2 g) were weighed carefully and placed in distilled water. At specified time points, the hydrogels were removed from the water and was drained carefully and weighed again. The swelling was calculated by the eqn (1).1
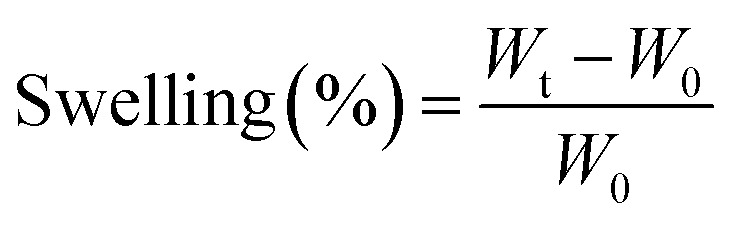
where *W*_t_ and *W*_0_ are the weights of the hydrogel after and before the swelling, respectively.

### 2.5 Determination of gel content

To assess the gel content, three dry gel samples were carefully weighed before being submerged in the swelling medium. Then, specified amounts of the dry hydrogel samples (*W*_b_) were immersed in deionized water in 250 mL flasks for 24 h. The swollen hydrogels were then cleaned and filtered repeatedly. The swollen samples were immersed in ethanol to eliminate water and then being dried at 50 °C in an oven for 24 h (*W*_a_). The gel content was estimated using eqn (2).2
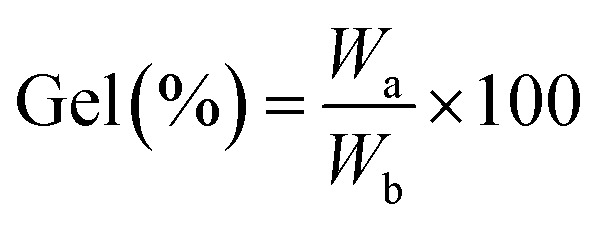


### 2.6 General experimental procedure for the synthesis of 1,2,3-triazoles

A glass vial was filled with 1.0 mmol of phenylacetylene, 1.5 mmol of sodium azide, 1.0 mmol of epoxide, 10–60 mg of CS/CuFe_2_O_4_ nanocomposite hydrogel as a catalyst, and 4.0 mL of distilled water. The heterogeneous reaction mixture was then stirred (250 rpm) for 12 h at room temperature. To investigate the development of the reaction, thin-layer chromatography (TLC) was used. After the starting materials were completely disappeared, 5.0 mL ethyl acetate was added into the reaction medium and stirred for 10 min before separating the nanocomposite hydrogel. This process was repeated three times. The extracted products were purified by column chromatography using silica gel and a mixture of ethyl acetate/hexane. Spectroscopic methods (FT-IR, ^1^H NMR, and ^13^C NMR) were applied to determine the structures of the products. Single crystal X-ray analysis was used to determine the crystal structure of one of the products (T2). The analysis of single crystal X-ray and structure refinements are present in the ESI†. A list of important crystallographic parameters related to single crystal X-ray analysis and refinement is depicted in Table 1.

**Table 1:** Structure refinement and crystal data for compound T2

Compound	T2
CCDC No	2264680
Net formula	C_13_H_17_N_3_O_2_
*M*_r_/g mol^−1^	247.29
Crystal size/mm	0.58 × 0.12 × 0.10
*T*/K	293
Radiation	Mo (0.71073 Å)
Crystal system	Monoclinic
Crystal shape, color	Needle/colorless
Space group	*P*2_1_/*c*
*a*/Å	8.6436 (5)
*b*/Å	18.9145 (12)
*c*/Å	8.3234 (5)
*β*/°	99.035 (6)
*V*/Å^−3^	1343.90 (14)
*Z*	4
Calc. density/g cm^−3^	1.222
*μ*/mm^−1^	0.09
*F*(000)	528
*θ* range/°	2.2–28.3
*h*	−11 → 11
*K*	−24 → 24
*l*	−4 → 10
*R*_int_	0.054
*R*1	0.0602
w*R*2	0.1184
*S*	1.041
Abs. correction	Multi-scan
Measured reflections	8914
Independent reflections	3043
Parameters	163
Restraints	0
Largest diff. peak and hole	0.418/−0.227

## 3. Results and discussion

### 3.1 Hydrogel synthesis and characterization

**Scheme 1:**
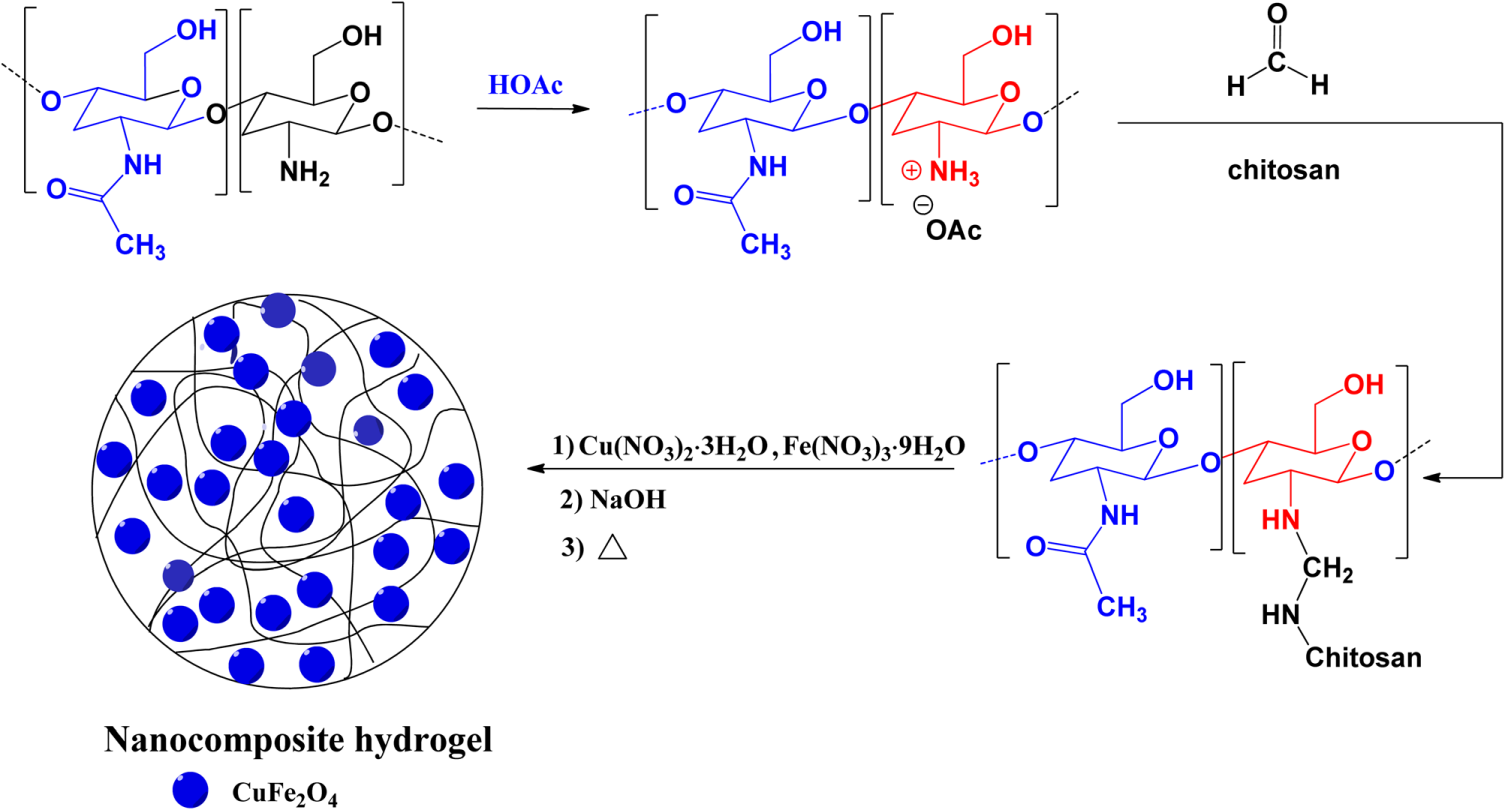
Schematic illustration for the formation of CS/CuFe_2_O_4_ nanocomposite hydrogel.

The hydrogel matrix consists of many micro and nano pores that can act as the nanoreactors. The nanoreactors can be utilized for production of the nanoparticles. The nanocomposite hydrogel was prepared by the reaction of chitosan and formaldehyde followed by treatment with Cu(NO_3_)_2_·3H_2_O and Fe(NO_3_)_3_·9H_2_O salts. Chitosan was cross-linked with formaldehyde by condensation of primary amines of chitosan with formaldehyde. Copper(ii) and iron(iii) ions also have a stabilizing role in hydrogel strength through ionic cross-linking. The cross-linking reaction and formation of the nanocomposite hydrogel are shown in Scheme 1. The nanocomposite hydrogel was synthesized by co-precipitation of copper(ii) nitrate and iron(iii) nitrate within the hydrogel in an alkaline aqueous solution. The structure of the CS/CuFe_2_O_4_ nanocomposite hydrogel was confirmed by FT-IR, TGA, DLS, FE-SEM, HR-TEM, EDS, and XRD analyses.

**Fig. 1:**
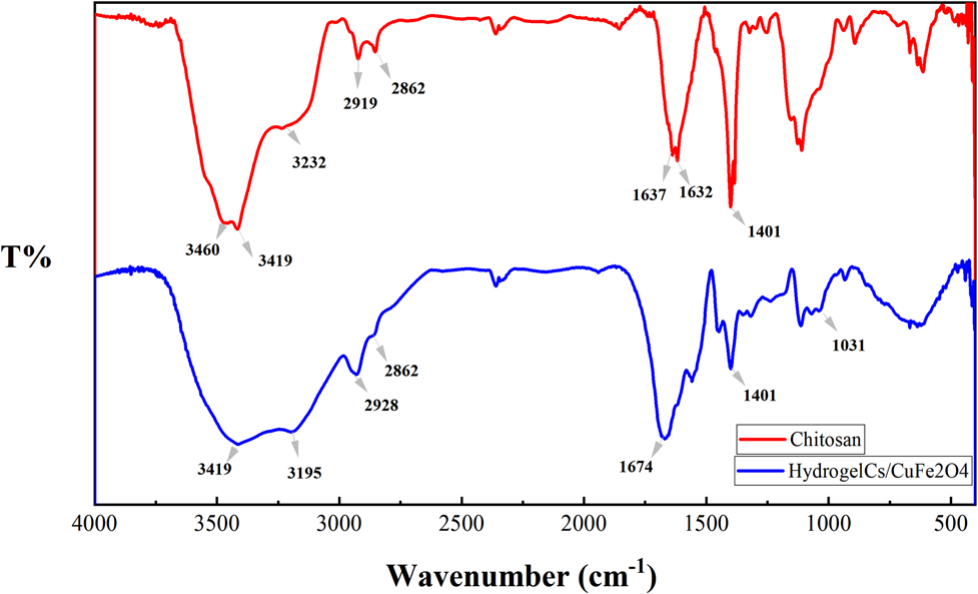
FT-IR spectra of chitosan (red line) and nanocomposite hydrogel CS/CuFe_2_O_4_ (blue line).

The FT-IR spectra of the pure chitosan and CS/CuFe_2_O_4_ nanocomposite hydrogel are shown in Fig. 1. The spectrum of hydrogel indicates the typical bands of the starting material with some discrepancies. The broad band with a center at about 3420 cm^−1^ is related to the stretching of the hydroxyl groups (O–H).^67^ The spectrum displays two well-defined peaks at about 3400 cm^−1^ which are also observed in chitosan. These peaks are somewhat weakened in the hydrogel sample, which is due to the reaction of the free amino groups with formaldehyde. In the spectrum of the hydrogel the remaining amide group of chitosan shows peak at around 1632–1637 cm^−1^,^68^ whereas the absorption band is shifted to 1674 cm^−1^ in nanocomposite hydrogel. The presence of CuFe_2_O_4_ in the nanocomposite hydrogel causes a shift in the peak. The change in absorption bands at about 1031–1171 cm^−1^ in the spectrum of hydrogel are due to the formation of new C–N bonds that are formed by reaction between amino groups and formaldehyde.^69^ These peaks are the result of asymmetric stretching of C–O–C and C–N–C units in the structure of the nanocomposite hydrogel.

**Fig. 2:**
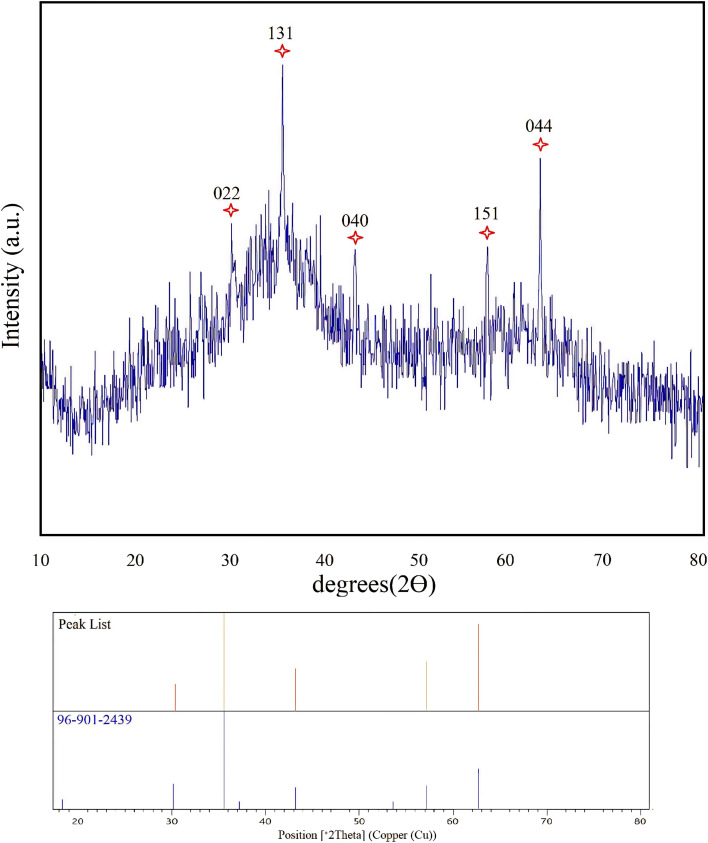
XRD pattern of nanocomposite hydrogel CS/CuFe_2_O_4_.

To investigate the presence of CuFe_2_O_4_ in the nanocomposite hydrogel, XRD analysis was performed at room temperature (Fig. 2). The XRD pattern demonstrated reflection peaks at 2*θ* = 30.32, 35.54, 43.18, 57.13, and 62.73° which are well in line with the standard XRD pattern of CuFe_2_O_4_ (96-901-2439). These results show the presence of CuFe_2_O_4_ in the composition of the obtained nanocomposite hydrogel. Also, the crystal size of CuFe_2_O_4_ nanoparticles was quantitatively calculated using the Scherrer equation, which was estimated to be about 46 nm.

**Fig. 3:**
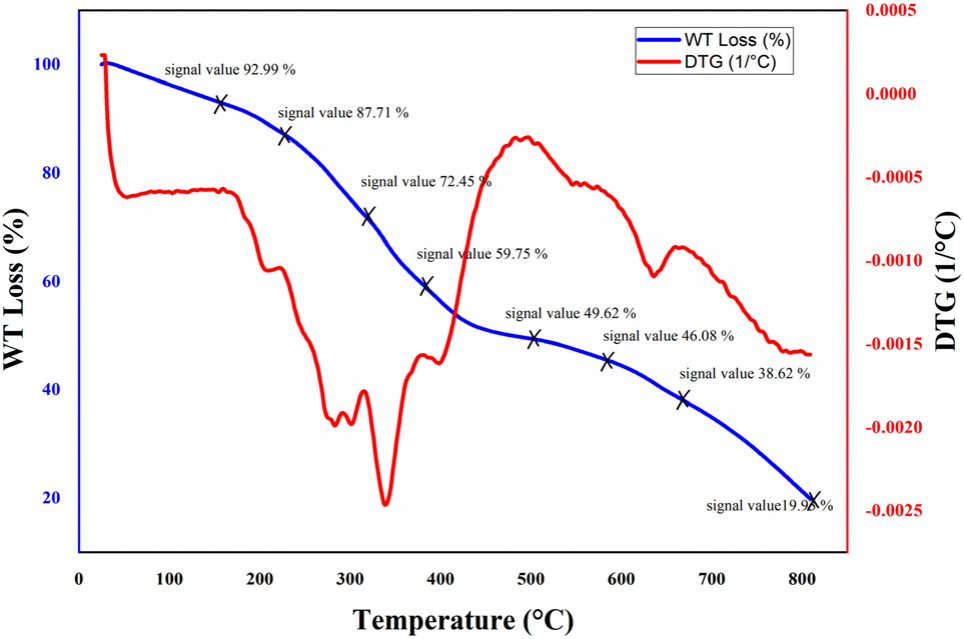
TGA analysis of the CS/CuFe_2_O_4_ nanocomposite hydrogel.

To investigate the thermal behavior of the nanocomposite hydrogel, thermogravimetric analysis (TGA) was conducted in the range of 25−800 °C (Fig. 3). TGA thermogram showed ∼7% of weight loss at 150 °C which is related to the removal of moisture from the structure. A gradual weight loss is seen at temperatures around 250 to 580 °C which is attributed to the degradation of the major backbone of the hydrogel. During this step, the polymer chains of the nanocomposite hydrogel are broken down into smaller pieces. Finally, when the temperature has reached about 750 °C, the residue ratio of the nanocomposite hydrogel was around 20%. This behavior indicates the remarkable heat stability of the synthesized nanocomposite hydrogel.

**Fig. 4:**
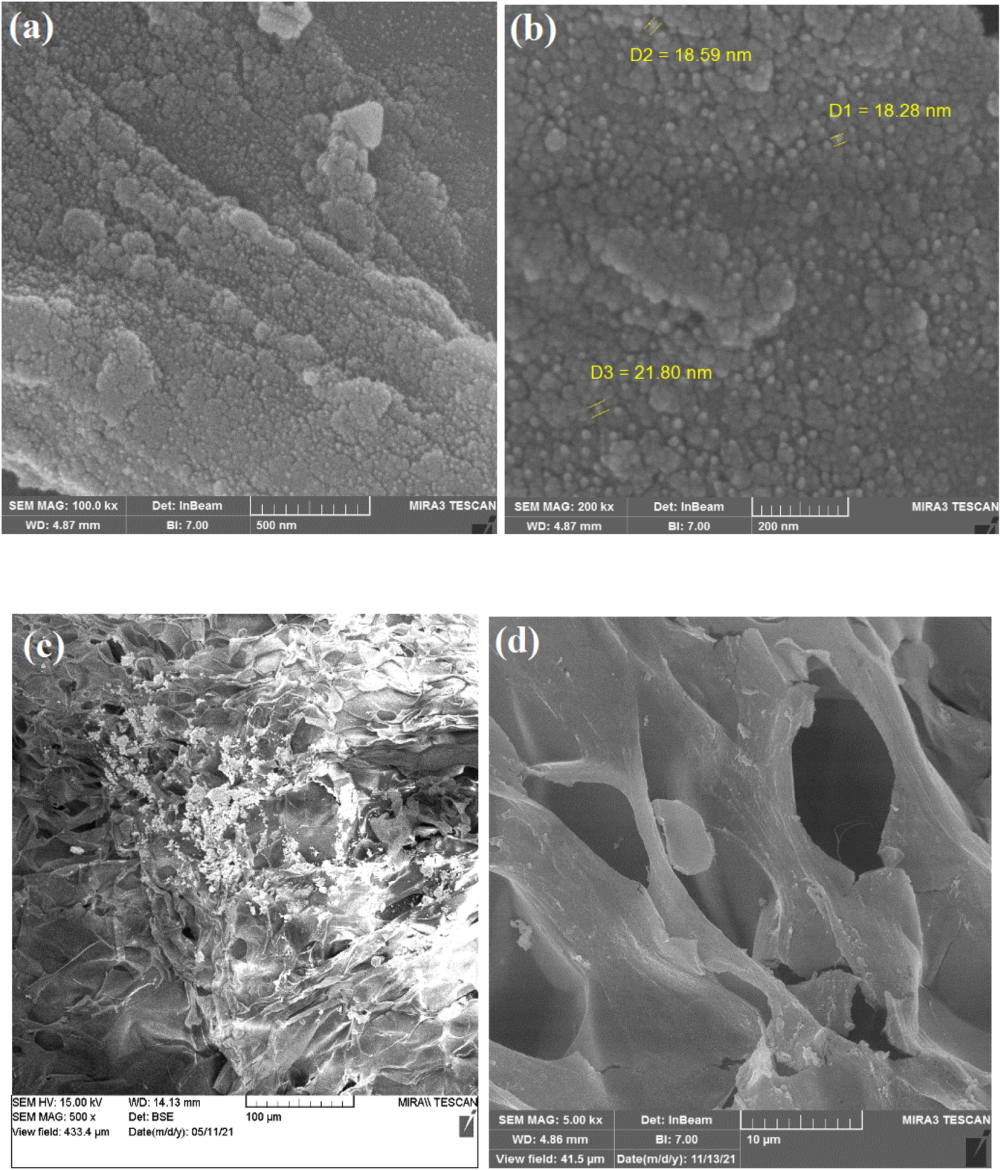
SEM analysis of the nanocomposite hydrogel catalyst (CS/CuFe_2_O_4_) with magnification of (a) 100 000×, (b) 200 000×, (c) 500× and (d) 5000×.

The field-emission scanning electron microscopy image (FE-SEM) technique was applied to study the surface morphology of the nanocomposite hydrogel. The nanocomposite hydrogel shows a rough and porous structure with a high number of holes. The CuFe_2_O_4_ nanoparticles with a pretty regular spherical shape are present on the surface of the catalyst, as shown in Fig. 4.

**Fig. 5:**
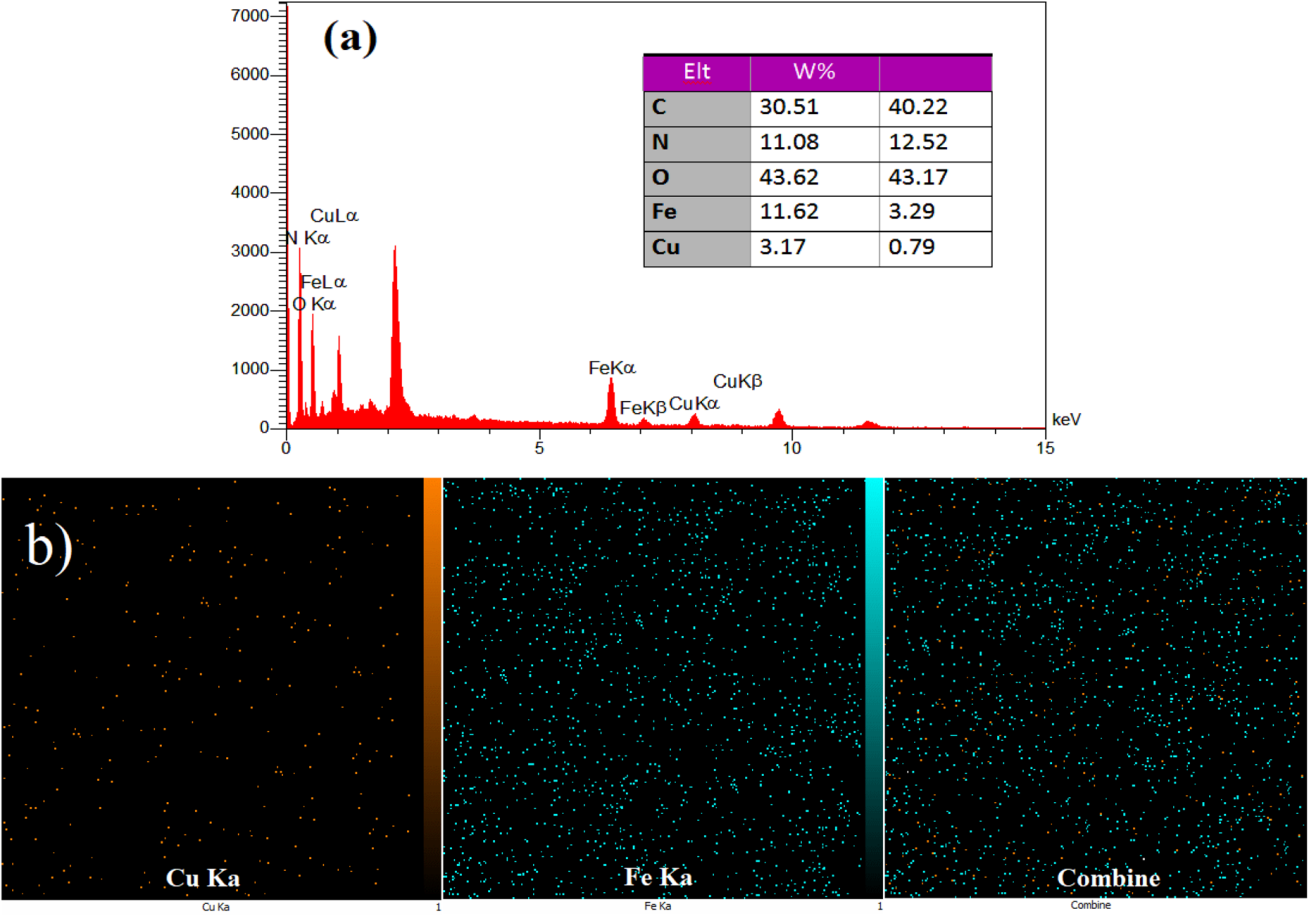
(a) EDS and (b) X-ray mapping of the CS/CuFe_2_O_4_ nanocomposite hydrogel catalyst.

EDS and X-ray mapping analysis were performed on the produced heterogeneous catalyst (see Fig. 5). The EDS spectrum (Fig. 5a) reveals the presence of copper (Cu) and iron (Fe) elements in the nanocomposite at percentages of 3.17 wt% and 11.62 wt%, respectively. In addition, the carbon (C, 30.51 wt%), oxygen (O, 43.62 wt%) and nitrogen (N, 11.08 wt%) are present in the composition of the synthesized catalyst which confirms the successful synthesis of nanocomposite hydrogel. EDS mapping was used to assess the distribution of Fe and Cu ions on the surface of the nanocomposite hydrogel. The Fe and Cu elements are uniformly distributed throughout the nanocomposite hydrogel (Fig. 5b).

**Fig. 6:**
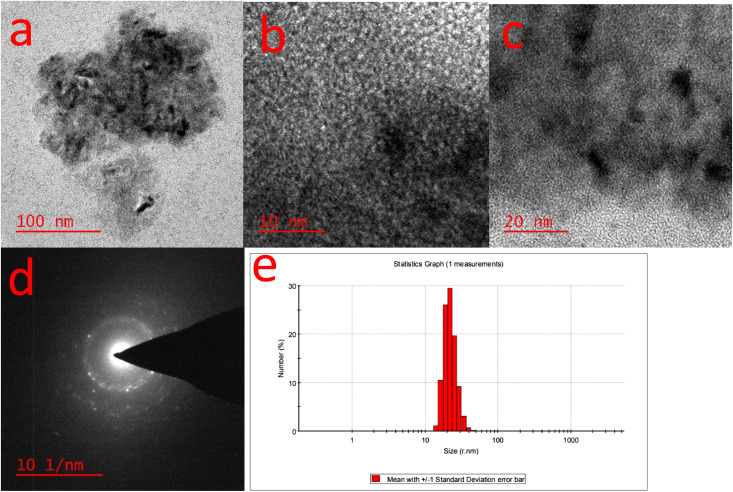
CuFe_2_O_4_ particle characterization by HR-TEM. HR-TEM image of the CuFe_2_O_4_ nanocomposite hydrogel catalyst, with various magnification (a–c), SAED pattern of the CuFe_2_O_4_ nanocomposite hydrogel (d), and particle size distribution obtained by DLS analysis (e).

The CuFe_2_O_4_ nanoparticles were studied by high-resolution transmission electron microscopy (HR-TEM) and DLS techniques. HR-TEM images are shown in Fig. 6 which reveal that the CuFe_2_O_4_ nanoparticles are present in the nanocomposite hydrogel with the size of about 5 to 20 nm. DLS analyses of the nanocomposite hydrogel show the hydrodynamic diameters of the nanoparticles with a particle size of about 18–30 nm, which can be confirmed by TEM. As expected, the size of CS/CuFe_2_O_4_ nanoparticles obtained from DLS analysis is higher than those obtained from TEM (Fig. 6).

### 3.2 Swelling rate and gel content

**Fig. 7:**
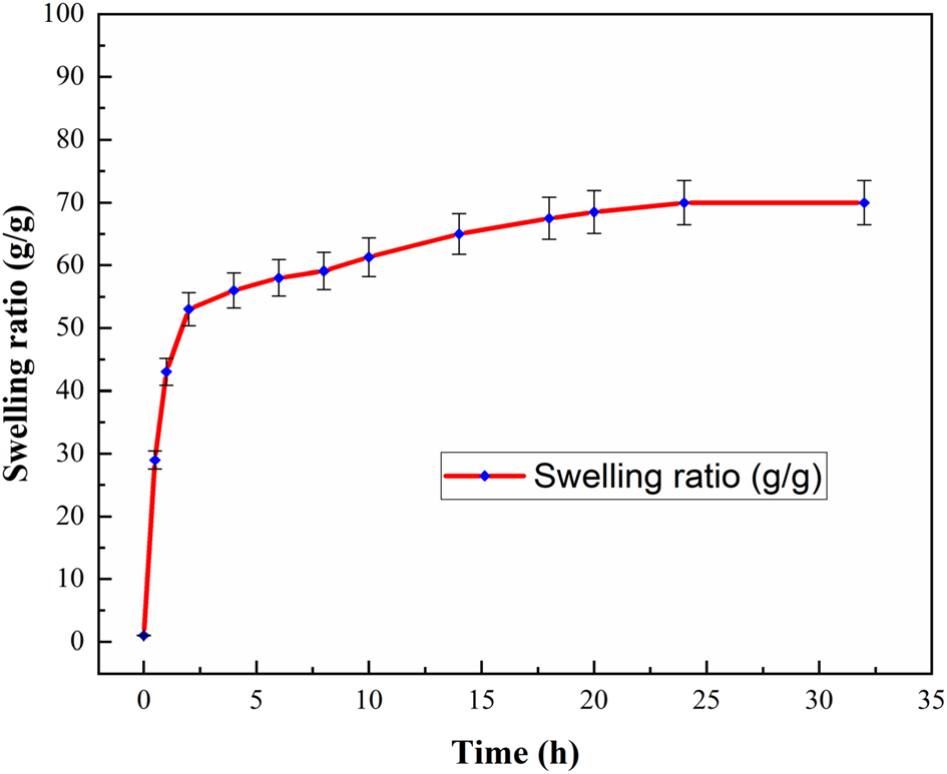
Swelling rate of CS/CuFe_2_O_4_ nanocomposite hydrogel at different times at 25 °C.

The swelling rate is an essential property of the hydrogels. The swelling behaviors of three samples of the nanocomposite hydrogels were studied at 25 °C in distilled water for 32 h. The averages of the swelling ratios were determined for the samples under the same circumstances and the results are shown in Fig. 7. It was found that the swelling rate of the nanocomposite hydrogels is sharply increased in the first two hours. Then, the rate of swelling showed a small change with time. The gel content of the nanocomposite hydrogel was also obtained 68.0%.

### 3.3 Catalytic activity

The catalytic ability of the CS/CuFe_2_O_4_ nanocomposite hydrogel was investigated in the synthesis of 1,2,3-triazoles in an aqueous solution at ambient temperature. As a model reaction, a combination of phenylacetylene, styrene epoxide, and sodium azide was selected. A specified amount of the synthesized CS/CuFe_2_O_4_ nanocomposite hydrogel was put in 4.0 mL of distilled water and shaken for 20 min. The reactive materials were poured in a reaction flask containing the catalyst and continuously stirred at 250 rpm. Thin-layer chromatography (TLC) was performed to track the course of the reaction. The optimization of the amount of the catalyst was carried out in the range of 10−60 mg and the results are collected in Table 2. The results demonstrate that an increase in the amount of catalyst led to a decrease in reaction time, which indicates that the catalyst possess a substantial effect on the rate of the reaction. The optimum amount of the catalyst was 40 mg for the production of 2-phenyl-2-(4-phenyl-1H-1,2,3-triazol-1-yl)ethan-1-ol with 96% yield at ambient temperature after 8 h. In the same conditions but without using catalyst, the reaction was not preceded even after 48 h.

**Table 2:** The effect of the amount of catalyst on the time of reaction and yield in the synthesis of 1,2,3-triazole (T1)

Entry	Catalyst (mg)	Time (h)	Yield (%)
1	0	48	0
2	10	21	48
3	20	15	76
4	30	11	94
5	40	8	96
6	60	7	95

**Fig. 8:**
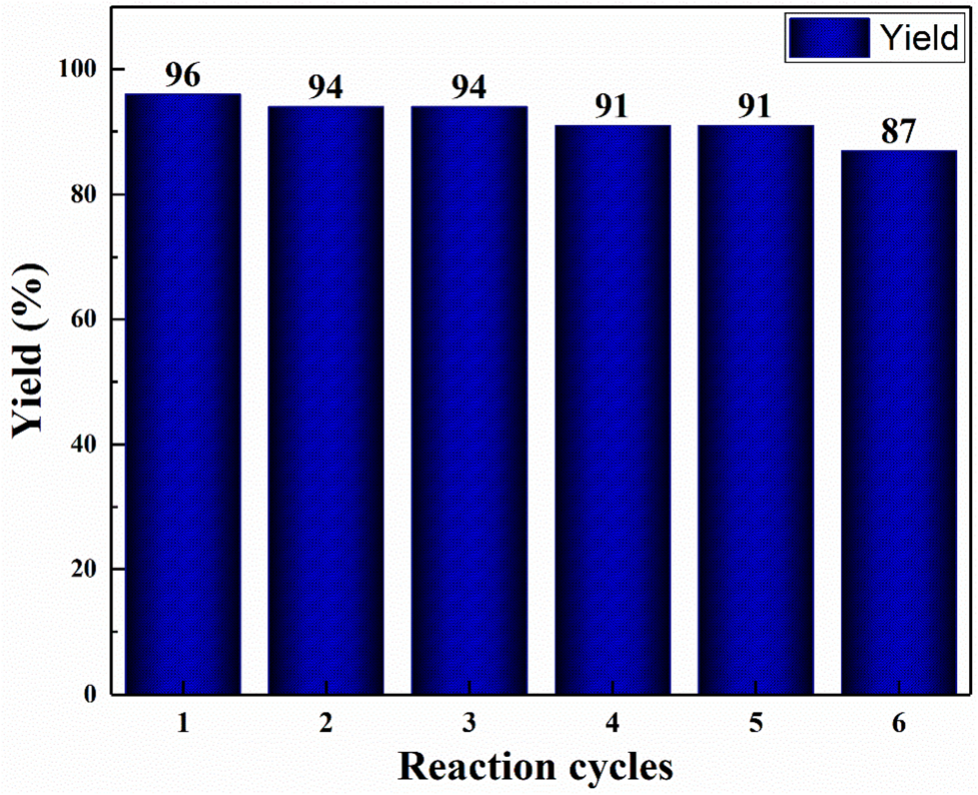
1,2,3-Triazole (T1) yield after each sequential reaction cycles.

To reduce the manufacturing costs, recycling of catalysts is an important factor. Therefore, reusability of the nanocomposite hydrogel catalyst was investigated. When the reaction was completed, 5.0 mL of ethyl acetate was poured to the reaction medium and then stirred for 3 min to separate the product from the catalyst. The catalyst was then removed from the reaction mixture, rinsed with distilled water, and used again in the same reaction. Six successive reactions were carried out with the nanocomposite hydrogel (Fig. 8). The catalyst remained stable even after six recoveries.

**Scheme 2:**
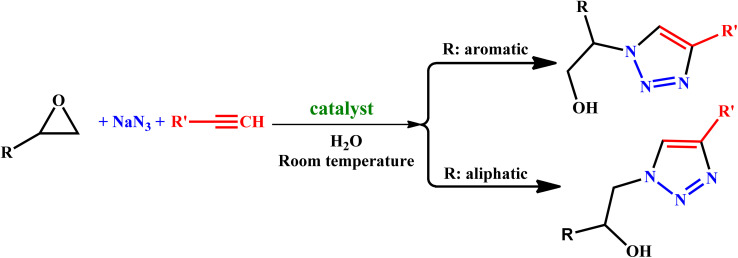
Synthesize of 1,2,3-triazoles using the CuFe_2_O_4_ nanocomposite hydrogel as a heterogeneous catalyst.

To examine the limitations of the reaction, a variety of derivatives of 1,2,3-triazole were produced by reaction of sodium azide with various epoxide and acetylenes (Table 3). High conversions (68–96%) were observed for all of these reactions, indicating the high catalytic activity of the CS/CuFe_2_O_4_ nanocomposite hydrogel for the synthesis of the triazoles. However, the reaction with aliphatic alkynes (2-methylbut-3-yn-2-ol, 1-methyl-2-cyclohexen-1-ol) was slower than aromatic alkyne (phenylacetylene), indicating the importance of the group attached to the alkynes in this catalytic reaction. These groups have also a considerable effect on the ring opening reaction (see Scheme 2), which has been discussed in detail in our previous reports.^70^

**Table 3:** The yields of the 1,2,3-triazoles synthesized using the nanocomposite hydrogel CS/CuFe_2_O_4_ as a heterogeneous catalyst^a^

Entry	Epoxide	Alkyne	Product	Time (h)	Yield (%)	Selectivity (%)
1	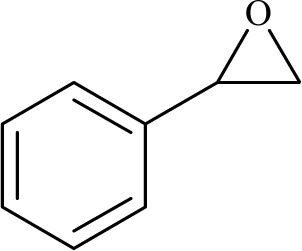	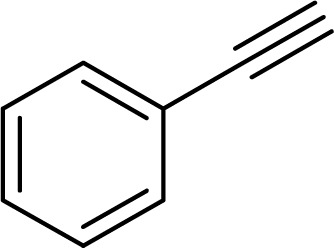	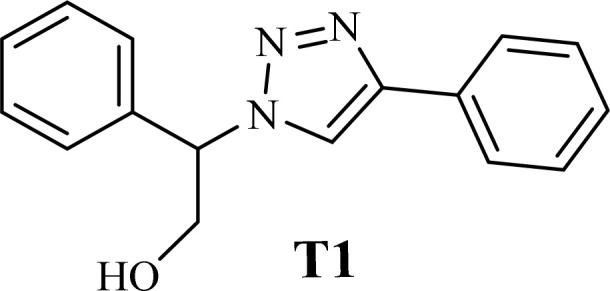	8	96	>99
2	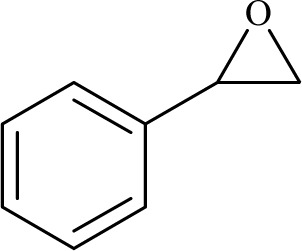	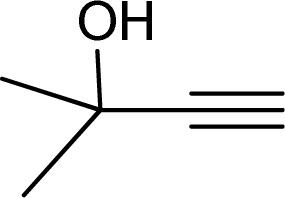	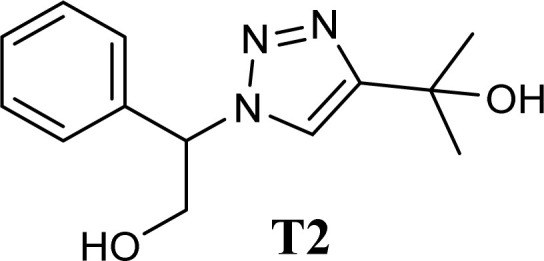	12	92	98
3	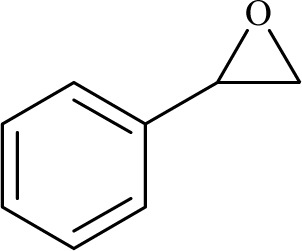	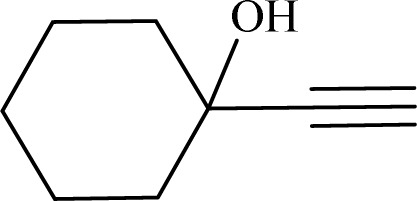	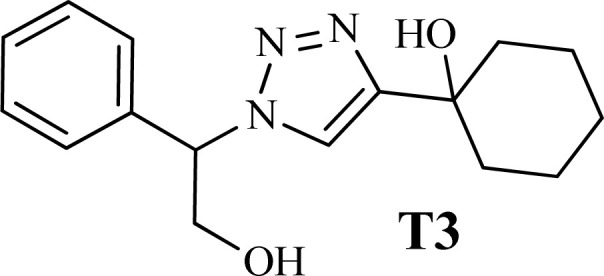	12	87	98
4	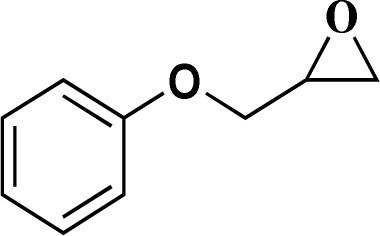	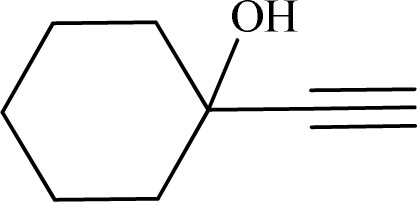	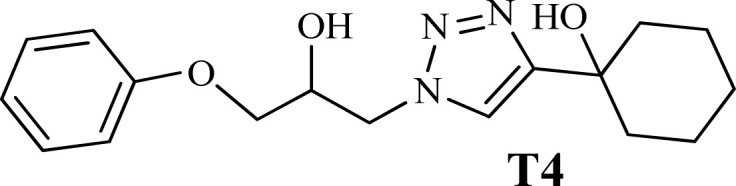	12	79	92
5	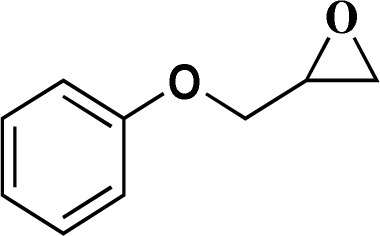	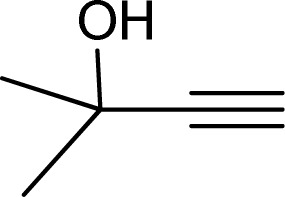	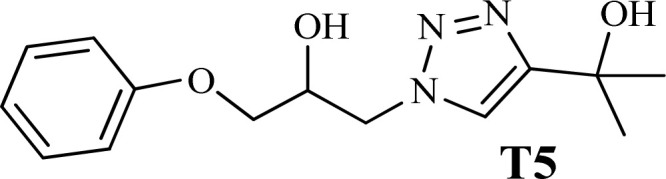	12	82	94
6	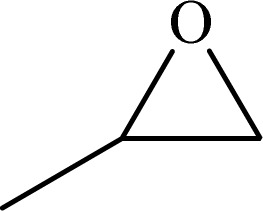	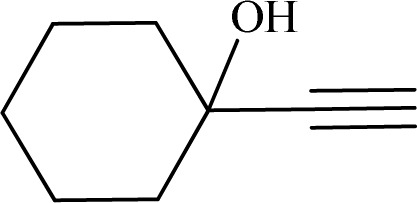	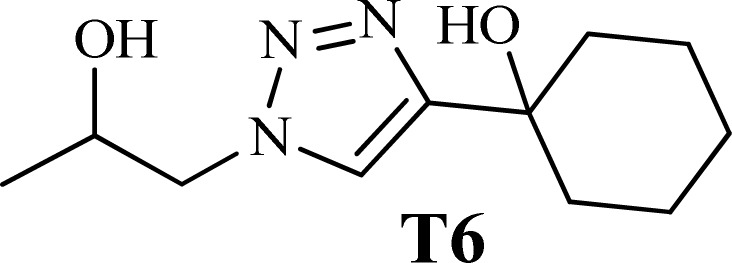	12	68	89
7	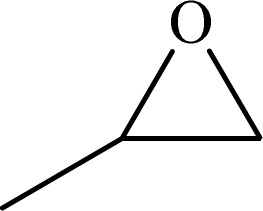	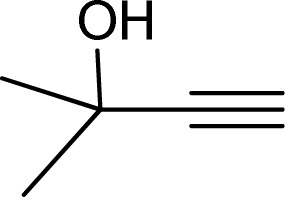	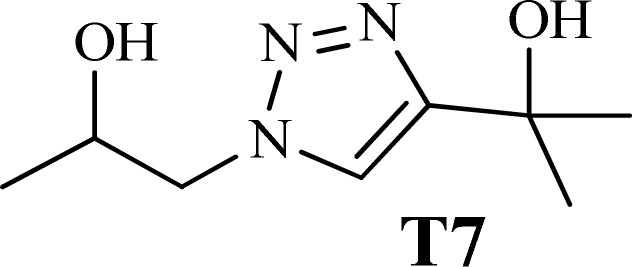	12	73	91

a Reaction condition: phenyl epoxy (1.0 mmol), alkynes (1.0 mmol), azide (1.5 mmol), nanocomposite hydrogel (40 mg of CuFe_2_O_4_ nanocomposite hydrogel) and 4.0 mL of H_2_O at room temperature and air atmosphere.

**Fig. 9:**
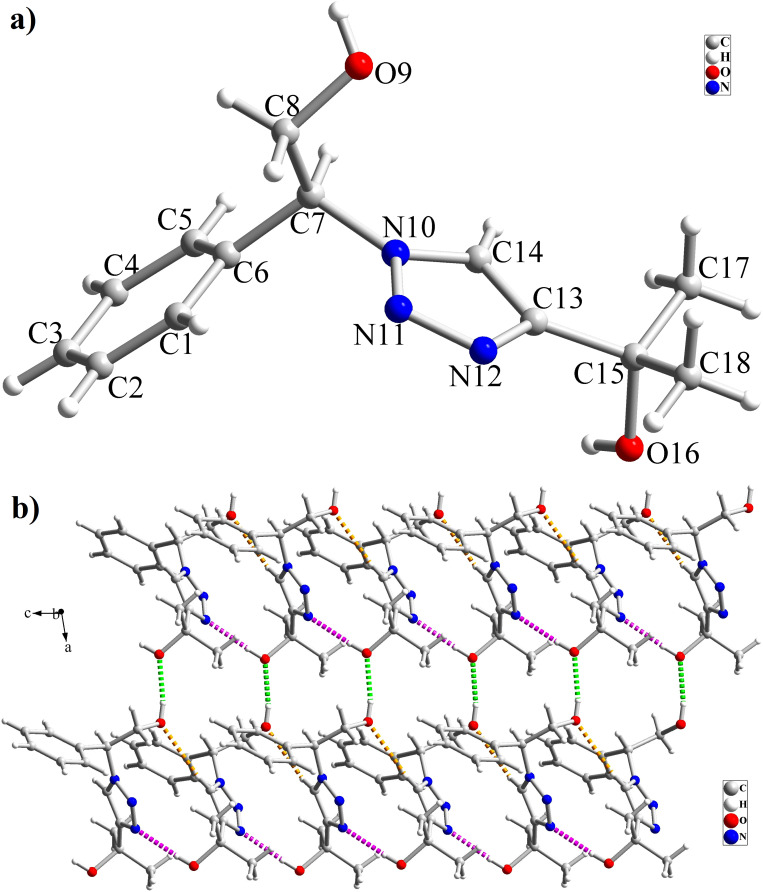
(a) Crystal structure of T2; (b) interactions in the structure of T2 are shown as green (O–H⋯O), pink (O–H⋯N) and orange (C–H⋯O) dashed lines.

The high purity products were obtained by recrystallization of the samples in methanol or ethanol solvents. All triazole derivatives were characterized by NMR and FT-IR spectroscopic methods after extraction and purification processes. In order to have stronger evidence about the formation of triazoles, the crystal structure of T2 was further studied using single crystal X-ray analysis. We have previously reported the crystal structure of this compound^71^ but, the primary results of the new data collection of the obtained crystals from methanol solvent indicated that the new crystals have different unit cell parameters than the previous one. After data collection and refinement processes, it was found that similar to the previous one^71^ the obtained crystals are crystallized in *P*2_1_/*c* space group but, it is a new polymorph of the compound. Although the product is the same as the previous, the new crystals have completely different intermolecular interactions which are consistent with the formation of a new crystal system. This situation is mainly related to the solvent that is used in the crystallization process. The structure of T2 is illustrated in Fig. 9a. The interactions of the crystal structure are shown in Fig. 9b. The important crystallographic data for T2 are shown in Table 1. The bond angles and bond lengths are also listed in Table S1.† The crystal structure of T2 showed that the formation of triazole was carried out by the ring–opening reaction of the epoxide from the more substituted position. In the structure of T2, two aromatic rings including phenyl and triazole are observed with an angle of 112.43° relative to each other. The intermolecular hydrogen bonds are demonstrated in Fig. 9b, which are formed by two alcoholic OH groups. In addition, C–H⋯π interactions are also present which are shown in Fig. 9b and S1.†

To demonstrate the ability of the catalyst, a comparison was made compared with those reported in the literature (see Table S3†).^70–74^ As indicated, the reaction times are comparable or slightly higher than that of the previous reports. Although the activity of the CuFe_2_O_4_ nanocomposite hydrogel catalyst is relatively lower than the corresponding homogeneous systems.^53,70,71^ The catalytic system exhibits great stability, significant activity, and good recyclability. In addition, the uniform dispersion of nanoparticles in the sponge-like hydrogel matrix provides a medium in which the valuable properties of the nanoparticles, including their high catalytic activity and large surface area, can be exploited.

The comparison of the synthesized chitosan/CuFe_2_O_4_ nanocomposite hydrogel catalyst with other catalysts demonstrates its suitable performance under green conditions (see Table 4). This catalyst achieves a high yield of 96% at room temperature after 8 h using water as the solvent, which is both environmentally friendly and energy-efficient compared to other catalysts that require higher temperatures or organic solvents. Furthermore, its catalytic activity was maintained over six successive cycles, showing its great reusability and cost-effectiveness. The combination of high efficiency, mild reaction conditions, and sustainability indicates the novelty and potential of the green and recyclable in practical applications.

**Table 4:** Comparing the catalytic performance of chitosan/CuFe_2_O_4_ nanocomposite hydrogel with various catalysts in the synthesis of 1,2,3-triazoles

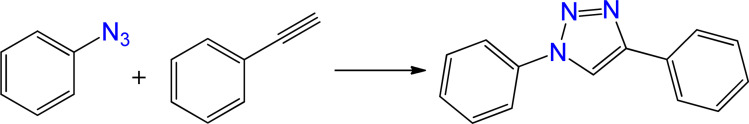						
Catalyst	Time (h)	Yield (%)	Temperature (°C)	Solvent	Reusability	Ref.
Cs/PVA-CuNP	6	92	50	*t*BuOH/H_2_O	70% after 5 cycles	64
Cu(ii)-AHG	24	92	Room temp.	H_2_O	62% after 4 cycles	63
Cu(ii)-AD	48	95	Room temp.	H_2_O	62% after 4 cycles	63
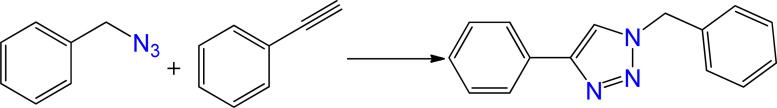						
CE-CD-Cu	24	96	40	H_2_O	6 cycles	75
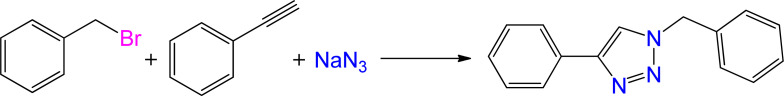						
Cu(i)@pNIPAM-VP	2	94	60	H_2_O	6 cycles	76
Cu(ii)@pNIPAM-VP	2	91	60	H_2_O	6 cycles	76
Cu(ii)@IPSi	24	91	Room temp.	H_2_O	6 cycles	76
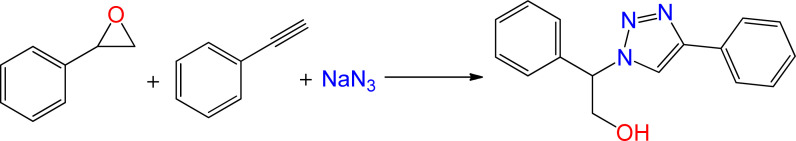						
Cu_2_O microspheres	24	95	Room temp.	H_2_O	Not specified	77
This work	8	96	Room temp.	H_2_O	87% after 6 cycles	—

## 4. Conclusions

In this study, a novel heterogenous catalyst for the synthesis of 1,2,3-triazoles was prepared from chitosan and formaldehyde, in the presence of Cu(NO_3_)_2_·3H_2_O and Fe(NO_3_)_3_·9H_2_O, using a simple two-step process. The process involved cross-linking of chitosan with formaldehyde, followed by *in situ* preparation of CuFe_2_O_4_ nanoparticles in the hydrogel network. The resulting CuFe_2_O_4_ nanocomposite hydrogel was characterized by XRD and EDX analysis. DLS and HR-TEM analysis indicated that the nanoparticles have the sizes of about 5–30 nm. The catalytic ability of the nanocomposite hydrogel was studied in the synthesis of 1,2,3-triazoles and the results indicated that the nanocomposite hydrogel can act as an effective catalyst for the cycloaddition reaction. In addition, the activity of the catalyst was maintained even after six cycles. The CuFe_2_O_4_ nanocomposite hydrogel can be introduced as an effective catalyst for green synthesis of triazoles, with easy synthesis, comfortable separation, and proper catalyst stability.

## Data availability

The data supporting this article have been included as part of the ESI.† Crystallographic data for the reported compound have been deposited at the Cambridge Crystallographic Data Center (CCDC-No. 2264680).

## Conflicts of interest

The authors declare that there is no conflicts of interest.

## Supplementary Material

RA-014-D4RA05626D-s001

RA-014-D4RA05626D-s002
