# Supplementary material for: Green and recyclable catalyst based on chitosan/CuFe2O4 nanocomposite hydrogel for one-step synthesis of 1,2,3-triazoles
Source: RSC Adv. 2024 Oct 1;14(43):31320–31. doi: 10.1039/d4ra05626d (PMC11443811; doi:10.1039/d4ra05626d)
Supplement: RA-014-D4RA05626D-s001 [file RA-014-D4RA05626D-s001.pdf]

Supplementary Information (SI) for RSC Advances.
This journal is © The Royal Society of Chemistry 2024

Electronic Supplementary Information

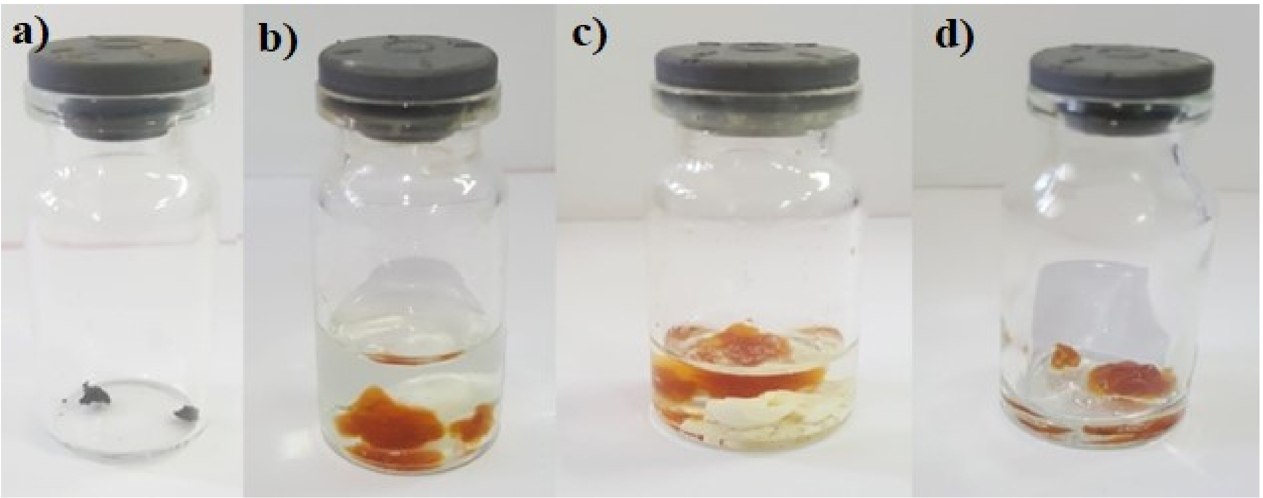

Fig. S1. Photographic images taken from the reaction system after each step; a) the catalyst in glass vial without the presence of azide and alkyne substrates and before swelling; b) the catalyst in the presence of azide and alkyne substrates in the beginning of the reaction; c) at the end of the cycloaddition reaction ; d) the catalyst exited and washed from the reaction mixture and also ready reused.

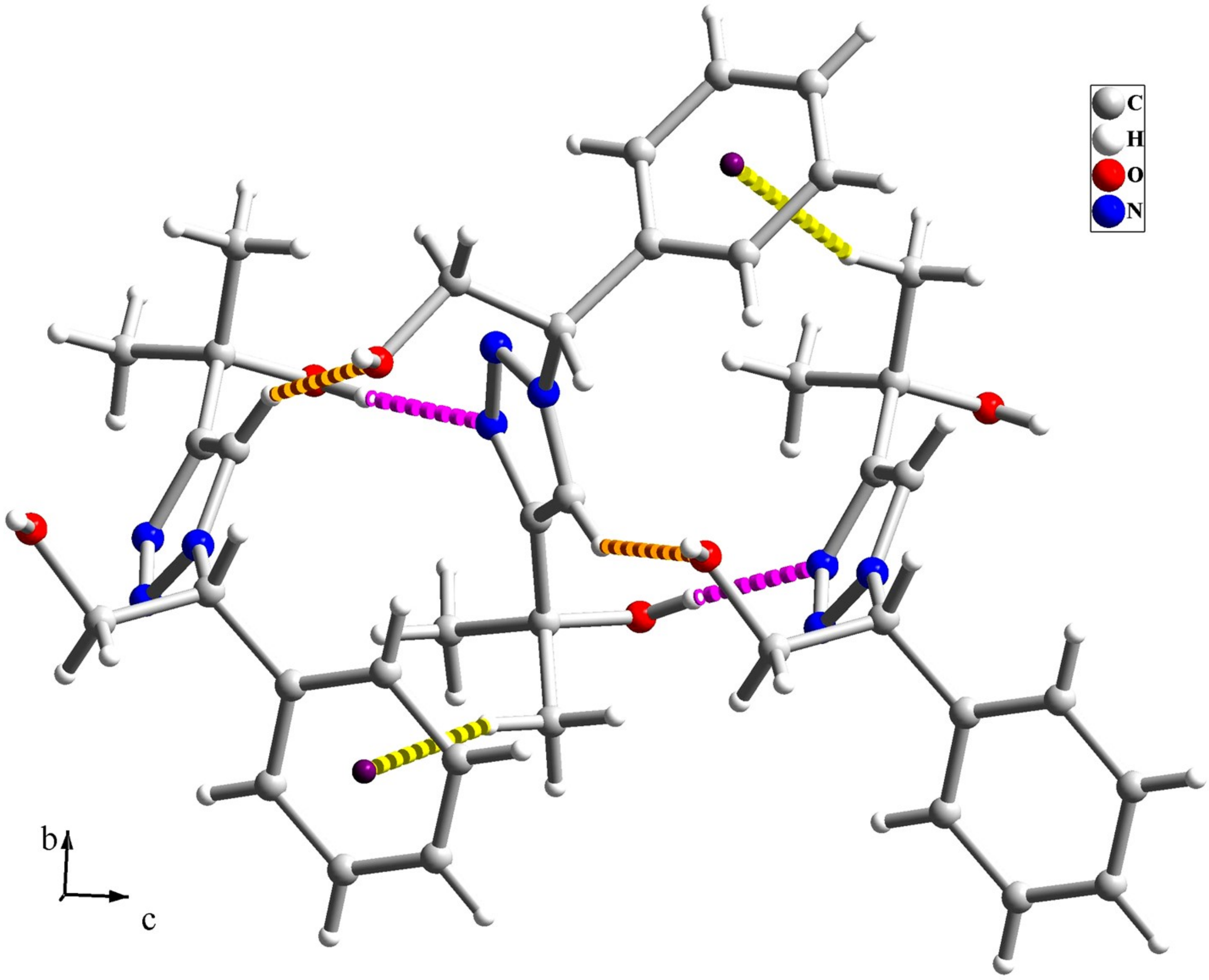

Fig. S2. Intermolecular O−H···N (pink dashed lines) and C−H···O (orange dashed lines) and C−H···π (yellow dashed lines) interactions in the crystal structure of **T2**

2-phenyl-2-(4-phenyl-1H-1,2,3-triazol-1-yl)ethanol (T1): M.p. 131-133 °C. *Anal.* Calc. for $C_{16}H_{15}N_3O$ (MW = 265.32): C, 72.43; H, 5.70; N, 15.84%. Found: C, 72.48; H, 5.73; N, 15.88%. FT-IR (KBr, cm^{-1}): 3393 (br-m), 3088 (w), 2925 (m), 2853 (w), 1604 (w), 1584 (w), 11482 (m), 1459 (s), 1382 (m), 1349 (w), 1306 (w), 1227 (w), 1223 (m), 1208 (m), 1187 (m), 1169 (w), 1152 (w), 1074 (s), 1050 (s), 1025 (s), 1000 (w), 976 (m), 959 (w), 910 (m), 850 (w), 826 (m), 761 (vs), 726 (s), 715 (w), 690 (vs), 663 (w), 550 (m), 512 (w), 493 (w), 479 (w). ^1H NMR (250.13 MHz, $CDCl_3$, 25 °C, TMS): δ = 7.75 (s, 1H), 7.71 (d, *J* = 6 Hz, 2H, Ar-H), 7.25-7.40 (m, 8H, Ar-H), 5.68 (dd, *J* = 8.13 Hz, *J* =3.75, 1H), 4.59-4.67 (2m, 2H), 3.80 (s, 1H). ^{13}C NMR (62.90 MHz, $CDCl_3$): δ = 147.65, 136.06, 130.18, 129.13, 128.97, 128.80, 128.26, 127.15, 125.65, 120.57, 67.32, 65.08 ppm.

2-(1-(2-hydroxy-1-phenylethyl)-1H-1,2,3-triazol-4-yl)propan-2-ol (T2): M.p. 117-120 ˚C. *Anal.* Calc. for $C_{13}H_{17}N_3O_2$ (MW = 247.13): C, 63.14; H, 6.93; N, 16.99 %. Found: C, 63.11; H, 6.95; N, 16.95%. FT-IR (KBr, cm^{-1}): 3395 (s), 3346 (s), 3334 (m), 3118 (w), 3064 (m), 3030 (w), 2982 (m), 2938 (w), 2901 (w), 2873 (m), 1602 (w), 1468 (m), 1456 (m), 1437 (w), 1378 (m), 1361 (m), 1335 (m), 1321 (w), 1273 (s), 1248 (s), 1225 (s), 1221 (s), 1198 (m), 1173 (s), 1157 (w), 1145 (m), 1115 (m), 1083 (m), 1050 (s), 1033 (s), 1012 (m), 971 (m), 957 (s), 906 (w), 862 (m), 844 (s), 748 (s), 735 (w), 729 (m), 692 (m), 680 (m), 636 (m), 623 (m), 615 (m), 607 (w), 543 (m), 508 (m), 493 (m). ^1H NMR (250.13 MHz, CDCl3, 25 °C, TMS): δ= 7.37-7.25 (m, 6H, N-CH=C, Ar-H), 5.59 (m, 1H, Ph-CH- CH2), 4.62-4.54 and 4.18-4.13 (m, 2H, -CH2-O), 3.41 (s, 1H, OH), 2.56 (s, 1H, OH) 1.61 (s, 6H, CH3). ^{13}C NMR (62.90 MHz, CDCl3): δ = 166.1, 129.1, 129.0, 127.1, 124.9, 120.1, 67.1, 65.2, 30.3 ppm.

1-(1-(2-hydroxy-1-phenylethyl)-1H-1,2,3-triazol-4-yl)cyclohexan-1-ol (T3): M.p. 88-91 ºC. *Anal.* Calc. for $C_{16}H_{21}N_3O_2$ (MW = 287.16): C, 66.88; H, 7.37; N, 14.62 %. Found: C, 66.79; H, 7.31; N, 14.74%. FT-IR (KBr, cm^{-1}): 3402 (br), 3272 (s), 3145 (s), 3089 (w), 3067 (w), 2933 (s), 2854 (m), 2100 (w), 1678 (m), 1656 (w), 1646 (w), 1636 (w), 1631 (w), 1604 (w), 1587 (m), 1497 (s), 1448 (s), 1388 (w), 1373 (m), 1352 (m), 1314 (m), 1278 (w), 1265 (m), 1252 (s), 1226 (s), 1177 (w), 1158 (s), 1132 (s), 1089 (s), 1073 (s), 1024 (m), 1005 (m), 1001 (m), 976 (s), 943 (m), 909 (s), 897 (m), 842 (w), 838 (s), 835 (w), 818 (w), 777 (w), 767 (w), 754 (m), 718 (w), 700 (s), 642 (m), 626 (w), 559 (w), 538 (m), 512 (w), 494 (w), 479 (w), 467 (w), 425 (w). ^1H NMR (250.13 MHz, $CDCl_3$, 25 °C, TMS): δ = 7.46 (s, 1H, NCH=C), 7.32-7.38-7.23 (m, 5H, Ar-H), 5.62 (s, 1H, OH), 4.48 (s, 2H), 4.01 (s, 1H), 3.21 (s, 1H), 1.84-1.27 (m, 10H, CH_2 cyclohexan). ^{13}C NMR (62.90 MHz, $CDCl_3$): δ = 155.1, 136.0, 129.8, 128.8, 127.1, 120.7, 69.3, 67.1, 64.8, 37.7, 25.3, 21.9 ppm.

1-(1-(2-hydroxy-3-phenoxypropyl)-1H-1,2,3-triazol-4-yl)cyclohexan-1-ol (T4): M.p. 107-110 °C. *Anal.* Calc. for $C_{17}H_{23}N_3O_3$ (MW = 317.39): C, 64.33; H, 7.37; N, 14.62 %. Found: C, 64.21; H, 7.32; N, 14.72%. FT-IR (KBr, cm^{-1}): 3389 (s), 3320 (s), 3155 (m), 3078 (w), 2928 (s), 2855 (m), 1633 (w), 1598 (s), 1589 (s), 1544 (w), 1499 (s), 1459 (m), 1447 (m), 1435 (m), 1383 (w), 1351 (w), 1339 (w), 1318 (w), 1295 (w), 1275 (m), 1244 (s), 1179 (m), 1161 (m), 1157 (m), 1136 (m), 1120 (w), 1081 (m), 1034 (m), 1068 (m), 1050 (s), 1039 (s), 996 (w), 977 (m), 958 (m), 930 (w), 906 (w), 897 (w), 887 (w), 875 (w), 849 (w), 829 (m), 819 (m), 748 (s), 692 (s), 612 (m), 510 (w), 406 (w). ^1H NMR (250.13 MHz, $CDCl_3$, 25 °C, TMS): δ = 7.58 (s, 1H), 7.26 (d, J = 4.2 Hz, 2H, Ar-H), 6.64 (t, 3H, J=5.7 and J=13.2, Ar-H), 5.02 (s, 1H), 4.64 (d, J = 9.5, 1H), 4.44 (s, 2H, CH_2), 4.00 (s, 2H), 3.66 (s, 1H), 1.84-1.29 (m, 10H, CH_2 cyclohexan). ^{13}C

NMR (62.90 MHz, $CDCl_3$): δ = 158.1, 154.8, 129.5, 121.9, 121.4, 114.5, 69.3, 68.9, 68.7, 53.4, 37.8, 37.6, 25.3, 21.9 ppm.

1-(4-(2-hydroxypropan-2-yl)-1H-1,2,3-triazol-1-yl)-3-phenoxypropan-2-ol (T5): M.p. 83-85 °C. *Anal.* Calc. for $C_{14}H_{19}N_3O_2$ (MW = 277.32): C, 60.63; H, 6.91; N, 15.15 %. Found: C, 60.52; H, 6.86; N, 15.25%. FT-IR (KBr, cm^{-1}): 3351 (s), 3315 (m), 3155 (m), 3064 (w), 2972 (w), 2930 (w), 2875 (w), 1598 (s), 1588 (s), 1546 (w), 1498 (s), 1468 (s), 1431 (w), 1399 (w), 1376 (m), 1359 (m), 1330 (w), 1292 (w), 1274 (s), 1276 (m), 1221 (m), 1174 (w), 1144 (m), 1111 (m), 1079 (w), 1062 (m), 1034 (m), 1020 (m), 958 (s), 925 (m), 884 (s), 858 (m), 755 (vs), 696 (s), 669 (m), 614 (w), 522 (w), 510 (w), 475 (w). ^1H NMR (250.13 MHz, $CDCl_3$, 25 °C, TMS): δ = 7.67 (s, 1H, NCH=C), 7.17 (d, *J* = 7 Hz, 2H, Ar-H), 6.85 (q, 2H, *J*=7 and *J*=7.5, Ar-H), 4.82 (2H, OCH_2), 4.57-4.33 (m, 1H, CH), 3.90 (2H, CH_2), 3.55 (1H, OH), 1.50 (s, 6H, $2CH_3$). ^{13}C NMR (62.90 MHz, $CDCl_3$): δ =158.20 (C-O, ArC), 155.16 (NCH=C), 129.55 (NC=CH), 121.81, 121.30, 114.50 (5× ArCH), 68.99, 68.51, 63.26, 53.23 ppm.

1-(1-(2-hydroxypropyl)-1H-1,2,3-triazol-4-yl)cyclohexan-1-ol (T6): M.p. 92-95 °C. Anal. Calc. for $C_{11}H_{19}N_3O_2$ (MW = 225.29): C, 58.38; H, 8.61; N, 18.61 %. Found: C, 58.64; H, 8.50; N, 18.65%. FT-IR (KBr, cm^{-1}): 3409 (m), 3085 (m), 3036 (m), 2957 (w), 2923 (w), 2890 (m), 1680 (vs), 1669 (s), 1637 (m), 1602 (s), 1592 (m), 1568 (m), 1495 (m), 1469 (m), 1453 (s), 1443 (m), 1436 (s), 1374 (m), 1342 (m), 1328 (w), 1311 (m), 1300 (w), 1272 (m), 1213 (w), 1157 (m), 1122 (s), 1079 (s), 1052 (vs), 1027 (m), 997 (s), 959 (m), 857 (s), 802 (vs), 755 (m), 734 (vs), 697 (w), 669 (m), 633 (w), 618 (m), 606 (m), 498 (m), 456 (s), 441 (s), 437 (w), 433 (m). ^1H NMR (250.13 MHz, $CDCl_3$, 25 °C, TMS): δ = 7.58 (s, 1H, Ar-H), 4.59-3.3.84 (m, 3H, CH_2-CH),

3.32 (s, 2H, OH), 1.84-1.27 (m, 10H, CH_2 cyclohexan), 1.12-1.04 (m, 3H, CH_3). ^{13}C NMR (62.90 MHz, $CDCl_3$): δ = 136, 120.79, 69.3, 67.1, 53.8, 37.7, 25.3,23, 21.9 ppm

2-(4-(2-hydroxypropan-2-yl)-1H-1,2,3-triazol-1-yl)propan-1-ol (T7): M.p. 87-89 °C. Anal. Calc. for $C_8H_{15}N_3O_2$ (MW = 185.12): C, 51.88; H, 8.16; N, 22.69 %. Found: C, 51.84; H, 8.15; N, 22.67%. FT-IR (KBr, cm^{-1}): 3386 (m), 3430 (m), 3155 (m), 2980 (w), 2925 (w), 2862 (w), 1705 (w), 1640 (m), 1589 (m), 1585 (m), 1404 (m), 1381 (m), 1338 (w), 1225 (m), 1172 (s), 1140 (s), 1056 (vs), 1019 (vs), 952 (s), 925 (m), 857 (s), 838 (s), 815 (m), 672 (w), 639 (m), 591 (w), 542 (w), 525 (m), 523 (w), 471 (m), 462 (w). ^1H NMR (250.13 MHz, $CDCl_3$, 25 °C, TMS): δ = 7.58 (s, 1H, Ar-H), 4.48 (m, 2H, OH), 4.29- 4.08 (m, 2H, -CH_2-CH), 3.75 (m, 1H, CH), 1.47 (s, 6H, CH_3), 1.18-1.10 (m, 3H, CH_3-CH). ^{13}C NMR (62.90 MHz, $CDCl_3$): δ = 155.1, 121.3, 68.6, 66.2, 57.0, 30.9, 19.5 ppm.

X-ray crystallography

The colorless needle crystals of **T2** were obtained from solvent evaporating method. The X-ray data for the reported structure was collected at 293(2) K with an Oxford Sapphire CCD diffractometer using Mo Kα radiation λ = 0.71073 Å and ω-2θ method. The structure was solved by direct methods and refined with the full-matrix least-squares method on F^2 with the use of SHELX2018 program packages. [1] The analytical absorption corrections were applied (CrysAlis version 171.38.43 package of programs [2] Rigaku OD, 2015). Structural data have been deposited at the Cambridge Crystallographic Data Centre: (CCDC No 2264680).

Table S1. Selected bond lengths and angles in the crystal structure of **T2**

**Bond**	**Lengths (Å)**	**Bond**	**Lengths (Å)**
C1—C2	1.380(4)	N10—N11	1.344(2)
C1—C6	1.384(3)	N10—C14	1.347(3)
C2—C3	1.372(4)	N11—N12	1.326(2)
C3—C4	1.372(5)	N12—C13	1.362(3)
C4—C5	1.396(4)	C13—C14	1.377(3)
C5—C6	1.387(3)	C13—C15	1.525(3)
C6—C7	1.531(3)	C15—O16	1.449(2)
C7—N10	1.485(3)	C15—C17	1.526(3)
C7—C8	1.498(3)	C15—C18	1.528(3)
C8—O9	1.423(3)		
**Bond**	**Angle (°)**	**Bond**	**Angle (°)**
C2—C1—C6	120.5 (3)	N11—N10—C14	110.82 (17)
C3—C2—C1	120.6 (3)	N11—N10—C7	121.23 (18)
C2—C3—C4	119.7 (3)	C14—N10—C7	127.94 (19)
C3—C4—C5	120.3 (3)	N12—N11—N10	106.86 (17)
C6—C5—C4	119.9 (3)	N11—N12—C13	109.38 (16)
C1—C6—C5	119.0 (2)	N12—C13—C14	107.45 (18)
C1—C6—C7	122.0 (2)	N12—C13—C15	122.67 (18)
C5—C6—C7	118.9 (2)	C14—C13—C15	129.9 (2)
N10—C7—C8	110.73 (19)	N10—C14—C13	105.48 (19)
N10—C7—C6	112.44 (17)	O16—C15—C13	109.35 (17)
C8—C7—C6	112.8 (2)	O16—C15—C17	109.43 (18)
O9—C8—C7	111.9 (2)	C13—C15—C17	110.66 (18)
O16—C15—C18	105.26 (18)	C13—C15—C18	110.62 (19)
		C17—C15—C18	111.4 (2)

Table S2. Intermolecular hydrogen bond interactions in the crystal structure of **T2**

D—H···A	D—H	H···A	*D*···*A*	*D*—H···*A*
O9—H9A···O16^{i}	0.82	1.97	2.764 (2)	165.5
C14—H14A···O9^{ii}	0.93	2.63	3.412 (3)	143.7
O16—H16A···N12^{ii}	0.82	2.07	2.888 (2)	174.5

Symmetry codes: (i) x−1, −y+1/2, z−1/2; (ii) x, −y+1/2, z+1/2.

Table S3 Comparison of catalytic activity of different catalysts on the model reaction (synthesis of **T1**)

Entry	Catalyst	Time	*Temp. (°C)*	*Yield (%)*	*Reference*
1	Cu(10%)/Meso-ZSM-5	6	70	94	[41]a
2	Cu(10%)/ZSM-5	6	70	54	[41]a
3	SG-APTS	3	50	93	[39]
4	CuNPs/C	8	70	93	[41]b
5	CuCl2	20	70	64	[41]b
6	NP-Cu	6	80	92	[41]c
7	$[Cu(H_3L)(\mu_{1,3}\text{-}N_3)(N_3)]_n$	3.5	40	93	[38]
8	$CS/CuFe_2O_4$	8	25	96	In this work

References

[1] G. M. Sheldrick. Acta Cryst.. 2015, C71, 3-8.

[2] CrysAlis 171.38.43 package of programs. Rigaku Oxford Diffraction, 2015.
